# A Bioinspired Cownose Ray Robot for Seabed Exploration

**DOI:** 10.3390/biomimetics8010030

**Published:** 2023-01-12

**Authors:** Giovanni Bianchi, Lorenzo Maffi, Michele Tealdi, Simone Cinquemani

**Affiliations:** Dipartimento di Meccanica, Politecnico di Milano, Via La Masa 1, 20156 Milano, Italy

**Keywords:** bioinspired robot, swimming locomotion, autonomous underwater vehicle, cownose ray, batoid fishes, flexible fins

## Abstract

This article presents the design and the experimental tests of a bioinspired robot mimicking the cownose ray. These fish swim by moving their large and flat pectoral fins, creating a wave that pushes backward the surrounding water so that the fish is propelled forward due to momentum conservation. The robot inspired by these animals has a rigid central body, housing motors, batteries, and electronics, and flexible pectoral fins made of silicone rubber. Each of them is actuated by a servomotor driving a link inside the leading edge, and the traveling wave is reproduced thanks to the flexibility of the fin itself. In addition to the pectoral fins, two small rigid caudal fins are present to improve the robot’s maneuverability. The robot has been designed, built, and tested underwater, and the experiments have shown that the locomotion principle is valid and that the robot is able to swim forward, perform left and right turns, and do floating or diving maneuvers.

## 1. Introduction

The development of autonomous underwater vehicles (AUVs) is a research field of great interest since the applications of AUVs are several, ranging from environmental monitoring and submarine exploration to aquatic farming and maintenance of infrastructure [1]. All these applications require staying underwater for a long time, maneuvering in narrow environments, and disturbing the living beings populating the seas as least as possible. Considering these requirements, conventional AUVs, propelled by helical thrusters, are less performant than fishes, as the propulsion mechanisms of the latter are characterized by great energy efficiency and maneuverability [1]. Therefore, the locomotion of fish is a promising source of inspiration for designing novel propulsion mechanisms for AUVs.

Fishes and cetaceans exhibit a wide variety of swimming strategies, which can be classified mainly into two categories: Body-Caudal Fin (BCF) swimming and Median Paired Fin (MPF) swimming [1,2]. BCF swimming is typical of the vast majority of fishes and involves the undulatory movement of the caudal fin and part of the body. Conversely, MPF swimming is characterized by the deformation of the pectoral or dorsal and anal fins creating a traveling wave that pushes water backward and generates thrust because of momentum conservation. BCF swimmers are characterized by high cruise speed and burst acceleration, whereas MPF locomotion is more advantageous in maneuverability and stability [1,2]. Mantas and rays belong to the last category as they swim flapping their large triangular fins, and their swimming strategy is considered as one with the highest energy efficiency and maneuverability [3]. Moreover, unlike thrust propellers, they do not disturb wildlife, so they are the best choice for marine life observation and environmental monitoring [4,5,6]. Biomimetic robots could also interact with fishes, so they are useful for studying the collective behavior of fishes [7,8,9,10], or to influence their behavior [11], making this kind of robot a useful tool to give fishes socio-emotional support [12].

These fishes belong to the order Batoidea and have dorsoventrally flattened bodies and large pectoral fins fused to the head that form a wide flat structure with the shape of a disc or a diamond [13]. The fin movement consists of the propagation of two waves on the fin, one traveling in the chordwise direction, i.e., from the head to the tail, and another in the spanwise direction, i.e., from the fin root to the fin tip. The main contribution to thrust generation is given by the chordwise traveling wave, which is responsible for pushing water backward, whereas the spanwise traveling wave is caused by the flexibility of the fin, and its effect is a delay in the motion of the fin tip which enhances the hydrodynamics [3] and helps to stabilize the locomotion by reducing the vertical force and the pitching moment [14]. The movements of different species are classified into two categories according to the ratio between the body length *L* and the wavelength λ of the chordwise wave. If the ratio L/λ is greater than 0.5, the movement is considered oscillatory; conversely, if smaller than 0.5, it is called undulatory [13]. The species adopting the undulatory locomotion are the smallest; they generally live near the seabed and cannot achieve high speed, but they have excellent maneuverability to perform turns with a null curvature radius [15]. The largest species, instead, move their fins in an oscillatory fashion since this allows them to gain more thrust and swim for a long time at high speed in pelagic environments, but they are less agile in maneuvers than the undulatory species [13]. Batoids fully occupy the continuum from completely undulatory to completely oscillatory swimming, and the species such as the cownose ray (*Rhinoptera bonasus*, Mitchill, 1815) lying in the middle of this continuum are featured by a good trade-off between speed and maneuverability. Cownose rays are featured by a ratio L/λ equal to 0.4, and they flap their fins at about 1 Hz achieving a cruising speed of ∼1.2 m/s [16].

These features have inspired the design of many biomimetic robots that swim like mantas or rays, and the challenge of mimicking such a complex movement has been addressed with several different solutions.

Some robots are moved by soft actuators, which have the advantages of being integrated into a completely soft and flexible robot without the limitations of rigid links and actuators and of making the actuation distributed over the whole fin, obtaining a fin movement very similar to the one generated by fishes. Electro-ionic actuators are adopted by the Fast Moving Electronic Fish, inspired by a batoid fish [17], and by the Soft Biomimetic Robot, inspired by a tuna [18]. The swimming performances of this robot are excellent in terms of speed and maneuverability, but this kind of technology is usually used only for robots of small dimensions (<10 cm).

The majority of the existing biomimetic robots, instead, are actuated by traditional motors and joints; nevertheless, excellent replication of the fin deformation and swimming performances can be achieved. A possible actuation solution is to use three or more independent mechanisms for each fin and replicate the traveling wave moving them with a phase delay. This is the strategy adopted by Mantabot, which actuates the fins with active tensegrity beams surrounded by a flexible elastomer [19], and by Roman III and Roman IV, whose fins consist of a thin silicone sheet with three ribs attached actuated by brushless motors [20,21]. The Bionic Fish uses three mechanisms actuated with a phase delay to recreate the traveling wave in each fin; these mechanisms are articulated so that they accurately reproduce the curvature of the fin [22]. Similarly, the Manta Ray Robot has fins actuated by an articulated mechanism actuated by two servomotors which recreate the curvature and the traveling wave on the fin [23]. The Manta Robot has three motors for each fin which give it excellent maneuverability thanks to the control algorithm based on phase oscillators [24]. Fins actuated by several mechanisms are also present in the Bionic Manta Ray Robot [25] and in the Robotic Manta [26]. The Novel Robotic Manta Ray uses eight Soft Fluidic Actuators to move the fin, the actuators have different cross-sections and lengths, and the traveling wave is reproduced by exploiting the different effects of the viscosity-induced resistance in different actuators [27]. Robots with fins actuated by several motors usually have extraordinary maneuverability since they can impose the traveling wave velocity and direction of the fin so they can perform turns with null curvature radius, and some can even swim backward. However, mounting many actuators on the fin imposes some limitations on the fin shape and material, which needs to be highly stretchable.

A different approach consists of modeling the fin’s oscillatory movement in the combination of a flapping movement and a pitching rotation of the fin properly phased, which is possible because, for the cownose ray, the body length is ∼0.4 times the wavelength [13]. These robots have only two degrees of freedom per fin, and only two motors are needed to actuate them, one for flapping and one for pitching. The Biomimetic Cownose Ray [28] and the Bionic Manta Ray Robot [29] have fins composed of flexible silicone ribs mounted on a flexible shaft and covered by an elastic skin; conversely, the Aqua Ray [30] and the Manta Ray AUV [31] actuate the fins with Bionic Fluidic Muscles allowing a large amplitude fin deflection. Another biomimetic robot exploiting this strategy to reproduce the fin deformation is the Bionic Robot Fish, which reproduces the fin shape very accurately and uses an articulated mechanism composed of sliding rods and spherical joints to achieve a flapping and pitching movement [32]. A quite similar mechanism is adopted by the Bionic Pectoral Fin, which deforms producing both a chordwise and a spanwise wave [33]. This approach is simple but very effective in generating thrust; however, it can be applied only to reproduce motions with large wavelengths.

Finally, many robots use a single actuator to move each pectoral fin, and the traveling wave is obtained passively, thanks to the fin flexibility. This working principle is exploited by several biomimetic robots inspired by different kinds of fishes. Some examples are the Underactuated Robotic Fish [34], the Robotic Tuna [35], and Tunabot Flex [36], inspired by a carangiform swimmer. The moving part of their bodies are divided into four modules, only the first is directly moved by an actuator, and the others, instead, are free to move, generating a traveling wave, which leads to a large movement of the tail. This is the working principle of Robo-Ray II which has silicone rubber fins actuated by pneumatic artificial muscles [37], and of Robo-Ray III having fins made of a thin rubber sheet with a reinforced leading edge [37]. Similarly, the MantaDroid has fins made of a thin PVC film with a more rigid leading edge made of ABS; its fins are actuated by servomotors near the head of the robot, which actuate the rigid leading edge, and they are attached to the rest of the body only at the leading edge [38]. The Manta Ray Robot adopts a similar design and actuates the fins with a crank-rocker mechanism connected to a servomotor [39], and the Robotic Cownose Ray has similar thin fins made of an ionic polymer-metal composite [40].

This kind of design has several advantages, the most evident is the simplicity of the mechanism actuating the fin, which does not impose any constraint on thickness and dimensions as there is no need to host articulated mechanisms inside the fin. Moreover, a thin fin with a root detached from the main body has higher propulsive efficiency than a fin attached to the body. A detached fin is more flexible, and its trailing edge performs a movement of greater amplitude, making the angle of attack of the fin larger. Although this causes a reduction in the total lift force acting on the fin, the generated force is directed more in the swimming direction, considerably reducing the vertical component of the force and, consequently, the required power. Therefore, despite a slight decrease in thrust with respect to fully attached fins, there is a significant increase in energy efficiency, which makes this type of fin interesting for AUV design [41]. Although these robots have lower maneuverability than the robots previously described, steers with small curvature radii and high angular velocities can be achieved.

The robot presented in this article takes inspiration from the cownose ray and has a rigid central body and flexible fins made of silicone rubber. Each fin is actuated by a servomotor that drives a link inside the leading edge, and the traveling wave is obtained with the passive deformation of the fin. In addition, a tail acts as a rudder actuated by two servomotors, which is used for maneuvers. The main objective of this research is to demonstrate the aforementioned advantages of this propulsion mechanism; thus, the article is mainly focused on the aspects related to the fin design and the characterization of the swimming performances. The fins designed for this robot give very accurately reproduce the geometry and the dynamics of real cownose ray fins thanks to the method adopted to reproduce their shape and to the material employed. Thus, these fins allow the robot to swim at high speed and with high maneuverability compared to other similar robots despite the simple design.

The rest of the article is organized as follows: in Section 2, the design of the robot is described; in Section 3, the results of the experimental tests are presented and discussed; finally, Section 4 is dedicated to the discussion and the conclusions.

## 2. Robot Design

### 2.1. Cownose Ray Geometry and Fin Kinematics

Batoid fishes’ bodies are wide and flat, and their cross-section is approximated as a symmetric airfoil. For the cownose ray, the best approximation is obtained with a NACA0020 [22].

**Figure 1:**
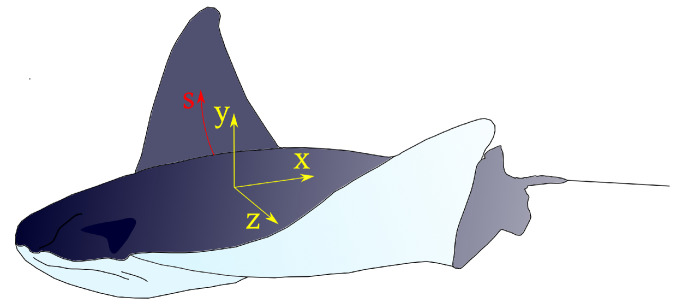
Representation of a cownose ray with the reference system adopted in Equation (1).

The kinematics of batoid fishes’ locomotion is derived from the experimental analysis by Russo et al. [16] who developed a biomechanical model of the fin, reconstructing its deformed shape at every time instant. In that work, the skeletal structure of the cownose ray was analyzed to quantify the parameters characterizing their fin motion. The cartilaginous structure of the fin is composed of several small radial segments connected with rotational joints. Considering the angle θ as the angle formed by a segment of the fin with the horizontal plane, θ of each segment is determined as follows:

(1) $$\theta \left(s,t\right)=\theta_{max}s\sin\left(\phi x+\psi s-\omega t\right)+\delta s$$

where $\theta_{max}$ is the angle at the fin tip, *s* is the distance of the cartilage segment from the fin root, *x* is the position from the leading edge, as shown in Figure 1, ϕ is the chordwise wave number divided by the fish body length, ψ is the spanwise wave number divided by the fin span, ω is the circular frequency of fin flapping, and δ is the mean value of the angle θ during a flapping cycle. The wave numbers ϕ and ψ are defined as follows:

(2) $$\phi =\frac{2\pi }{\lambda_{x}}\;\psi =\frac{2\pi }{\lambda_{s}}$$

where $\lambda_{x}$ is the wavelength in a longitudinal direction, whereas $\lambda_{s}$ is the wavelength along the fin span. Since the fin is composed of hundreds of small segments and joints [16,42], and the material of the fin is highly flexible, it is possible to consider the fin motion as a continuous deformation without losing accuracy in the representation of its geometry, and Equation (1) can be used to describe all the possible motions of a cownose ray fin.

Therefore, to obtain an accurate replication of fin locomotion, the central body should be rigid and with a hydrodynamic shape. The fins, on the other hand, should be very flexible, and spanwise and chordwise traveling waves should be present. This can be obtained by actuating the leading edge of the fin and leaving the fin tip and the trailing edge free to deform.

### 2.2. Robot General Description

The design of the robot’s central body and its fins are independent of each other, as each pectoral fin is actuated by a servomotor positioned in the front part of the robot, and the motor shaft is accessible to make it easy to change the fins. This design allows using the robot as a test bench for experiments on the efficiency of different fins in future research. Furthermore, the robot is equipped with two caudal fins, actuated independently by servomotors, which control the robot’s pitching rotation, ensuring the locomotion’s stability. The robot is neutrally buoyant, and its mass is balanced by adding ballasts. The fins should be moved at about 1 Hz with a maximum amplitude of ±45°, so the required velocity is at least 5 rad/s. To control the attitude of the robot, measurement of acceleration and angular rate are needed, so an IMU should be added to the robot.

### 2.3. Central Body Design

**Figure 2:**
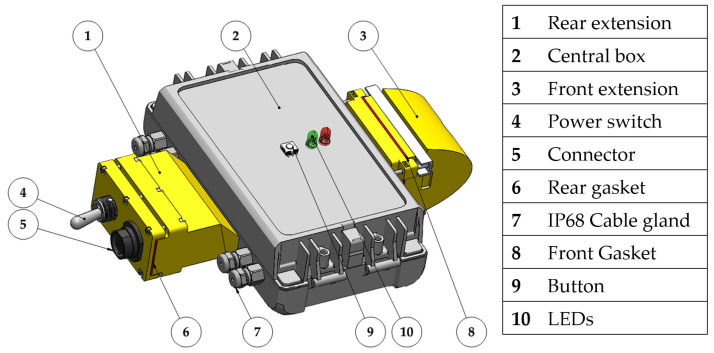
CAD model of the robot’s central body.

The robot’s central body is the housing for all the electronic components, so it is entirely waterproof and IP68 rated. It is composed of a main waterproof box with two 3D-printed extensions attached, as shown in Figure 2.

The box dimensions are 80 mm × 150 mm × 60 mm, and they have been selected to fit as tight as possible all the electronic components stacked between plastic layers inside the box. There are two LiPo batteries (Grepow GRP6134060) placed at the bottom, they are connected in series, and their nominal voltage is 3.7 V; their capacity is 1200 mAh, and the discharge rate is 15 C. Above the batteries, the Arduino Due electronic board is positioned. On the top level, all the sensors and accessories needed to control the robot and interact with it are placed; in particular, there are:

- Inertial Measurement Unit (IMU): the IMU is used to reconstruct the robot orientation in space; the chosen module is the GY-MPU9250, which includes a three-axis accelerometer, a three-axis gyroscope, and a three-axis magnetometer.
- Wi-Fi module: although it is not possible to communicate wireless underwater, it is still useful to equip the robot with a Wi-Fi connection to communicate data and change the control parameters without the need to directly access the board or the connector, which are sealed to avoid water leakage inside the box. The selected Wi-Fi board is the ESP8266-01, programmed with the ESP-link firmware, which creates a web server with a serial console from which it is possible to communicate with Arduino.
- SD-card module: it is used to store the navigation data since it is impossible to send them to the computer wireless in real-time while the robot is underwater.
- Ammeter: the ammeter is used to monitor the current delivered by the batteries and to evaluate the power consumed by the robot in the testing phase.

In this first configuration, for the preliminary tests, a pressure sensor is not present on the robot, but it will be used for future tests when a depth control is implemented. The selected pressure sensor is the BAR02-SENSOR-R2-RP, IP68 rated, which can be installed at the bottom of the robot on the aluminum chassis.

The rear extension of the box contains a switch that has remarkable dimensions because it is IP68 rated and an IP68 rated seven-pin connector that is used to recharge and balance the batteries and connect to the electronic board. The front extension contains a camera module OV7670 used to capture images from the robot’s point of view while swimming, which at this stage has no role in the control of the movement.

**Figure 3:**
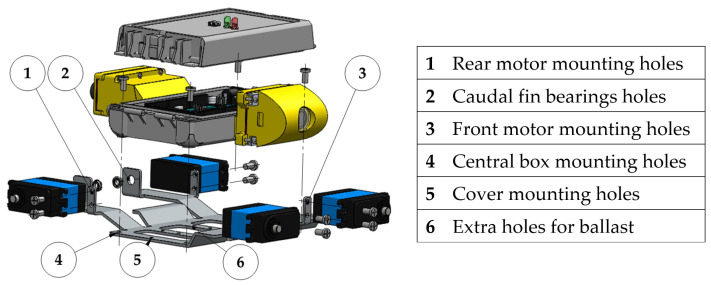
Exploded view of the assembly highlighting the components on the chassis.

The box is mounted on a chassis formed by a 2 mm thick aluminum sheet appropriately cut and bent, with holes and flaps to mount the box and the servomotors, as shown in Figure 3.

The servomotors are placed outside the box directly in contact with water, so they must be waterproof. The selected motors are digital brushless servomotors PowerHD 40 waterproof, IP68 rated, which have a stall torque of 3.9 Nm and a nominal speed of 12.4 rad/s when powered at 7.4 V.

**Figure 4:**
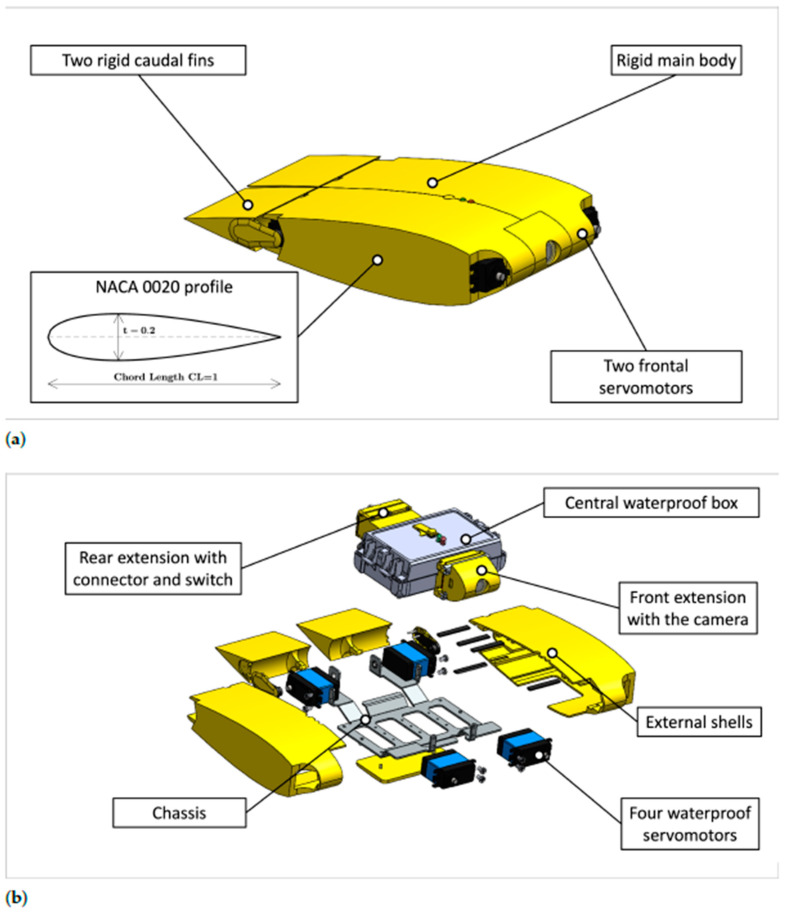
CAD model of the robot. (**a**) CAD model of the robot with the external shell; (**b**) Exploded view of the robot.

The central part of the robot is covered with a 3D-printed external shell to make its surface smooth and its shape more hydrodynamic. The cownose ray’s body can be approximated with a symmetric airfoil [22], and a NACA 0020 profile has been chosen for this robot. The thickness of the profile remains constant for the whole central body’s width. The caudal fins are rigid, and their shape corresponds to the trailing edge of the NACA 0020 profile constituting the central body tapering towards the trailing edge. Each caudal fin is connected to the servomotor by a bracket, and it is supported on the other side by a bearing mounted on the chassis. The allowed angle of rotation of the caudal fins is $\pm 45^{\circ }$. A CAD model and an exploded view of the robot assembly are shown in Figure 4.

### 2.4. Fin Design

**Figure 5:**
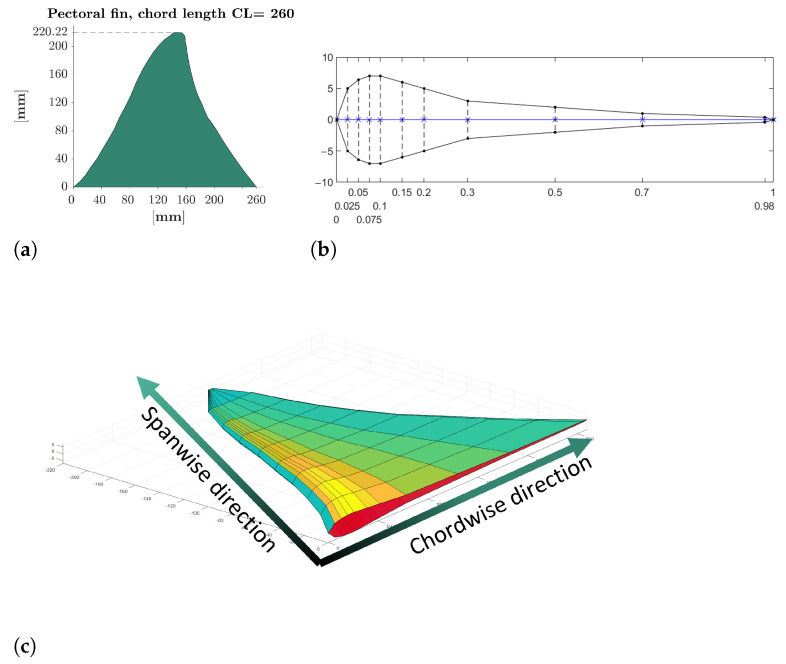
Fin geometry reconstruction. (**a**) Contour of the fin; (**b**) Cross-section of the fin; (**c**) Model of the fin.

The pectoral fins of the robot reproduce as accurately as possible the shape of a real cownose ray’s fins whose contour has been taken from the literature [22,43], and it has been scaled to the actual chord length of the robot, as shown in Figure 5a. The cross-section of the fin is a biomimetic profile that appears thicker near the leading edge and becomes thinner in the rear part arriving at the trailing edge almost flat because a fin with this shape, in the frequency range of the cownose ray, generates considerable more thrust than a fin shaped like a symmetric NACA profile [44]. The leading edge tapers toward the fin tip too. Combining the external contour and cross-section makes it possible to obtain the outer surface of the fin shown in Figure 5.

The fin is made of silicone rubber, and the leading edge is made stiffer by adding an aluminum stick mounted on the motor bracket. The fins are realized by molding, the liquid silicone rubber is poured inside 3D-printed molds, and it is vulcanized at room temperature.

The stiffness of the fin should be tuned to have the first natural frequency at about 1 Hz, in the frequency range where large amplitude movement is feasible with the selected motors. An accurate calculation of the vibration modes underwater is highly complex and far beyond the scope of this research, as it would require the coupled use of Computational Fluid Dynamics and Finite Element Analysis. Nevertheless, to understand if the designed fins deform in the desired way during their movement in the water, a simplified approach can be adopted [45]. This approach is based on the existence of a constant ratio between the natural frequencies of a body immersed in the water and the natural frequencies in the empty space, which is called Λ and is defined as:

(3) $$\Lambda =\frac{frequency\;of\;the\;mode\;in\;the\;water}{frequency\;of\;the\;mode\;out\;of\;the\;water}.$$

The natural frequency can be written as:

(4) $$f=\frac{1}{2\pi }\sqrt{\frac{k_{m}}{m_{m}}},$$

where $k_{m}$ and $m_{m}$ are the modal stiffness and the modal mass of the considered vibration mode. Since the stiffness of the fin does not change with immersion in the water, the frequency reduction ratio can be expressed as:

(5) $$\Lambda =\sqrt{\frac{m_{m}}{m_{m}+m_{w}}},$$

where $m_{w}$ is the added mass of water contributing to the vibration mode by increasing the inertia force acting on the fin, the ratio varies among vibration modes, and it is about 0.6 for the first natural frequency and tends to one as frequency tends to *∞*; the mode shapes, instead, are very similar inside and outside of water [45]. Assuming that this approach is also valid for the large deformations occurring for the fins, it is possible to calculate the first natural frequency of the fins out of the water and multiply them by this scale factor to obtain the natural frequency underwater.

The fins’ natural frequencies and vibration modes can be computed using the FEA software Abaqus, and it is necessary to consider the non-linearities due to the geometry and the material. The material of the fins is silicone rubber, which has a density of 1170 $\mathrm{kg}/m^{3}$, and a Young modulus *E* calculated using the following empiric formula [46]:

(6) $$S=100\mathrm{erf}\left(3.186*10^{-4}\sqrt{E[\mathrm{Pa}]}\right).$$

This equation is valid for rubbers with an A-shore hardness *S* higher than 40, which is the case of the rubber used for the fins, which has an A-shore hardness of 45, and the resulting Young modulus is 1.76 MPa. The material is modeled as incompressible and isotropic with a Neo-Hookean constitutive equation:

(7) $$W=C_{1}\left({\bar{I}}_{1}-3\right)$$

where *W* is the strain energy density, ${\bar{I}}_{1}$ is the first invariant of the left Cauchy–Green deformation tensor, and $C_{1}$ is a constant of the material which for silicone rubber is equal to 1.3078 MPa [47].

**Figure 6:**
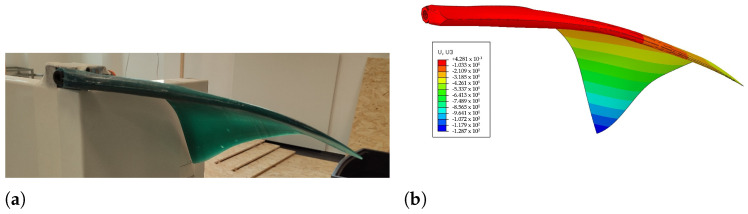
Comparison between experimental and numerically computed fin deflections. (**a**) Experimental; (**b**) Numerical.

To assess the correctness of the computed Young modulus and constitutive law, a static simulation of the deformation of the fin has been carried out, where the only load present is gravity. The results have been compared with experimental measurements, as shown in Figure 6, where it is possible to note that the difference between the results is minimal, as the numerically computed fin tip displacement is 128.7 mm, and the measured one is 123.7 mm.

Then, the frequency response of the fin is evaluated in two steps: first, a linearized frequency analysis is performed to have an approximate value of the first natural frequency, then some dynamic simulations using an implicit solver are carried out. In these simulations, a sinusoidal movement of the aluminum stick is imposed, and the fin deformation is computed. The analysis is repeated for different frequencies in the neighborhood of the natural frequency resulting from the first linearized step so to compute the frequency for which the trailing edge displacement is maximum. This frequency results equal to 1.35 Hz, which means that the first natural frequency underwater is 0.8 Hz. Thus, the selected motors can move the fins at the resonance frequency with a peak-to-peak amplitude of more than 90°.

A numerical investigation about the fluid dynamics of this locomotion strategy has been done in previous work [42], which described the results of some CFD simulations of a swimming cownose ray. This analysis showed that this kind of movement has great energy efficiency and that the Strouhal number is ∼0.3, as it was observed for real cownose rays and for the majority of fishes [48].

### 2.5. Robot Assembly

**Figure 7:**
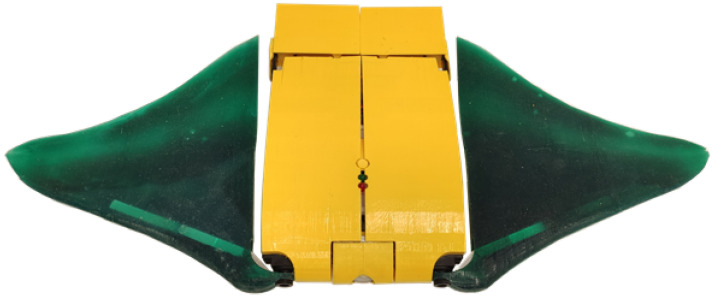
Assembly of the robot.

The robot has been built and assembled, as presented in Figure 7.

The total length of the robot is 260 mm, the full width, including the fins, is 620 mm, and the maximum thickness is 78 mm. The robot is made neutrally buoyant with the addition of ballasts between the central body and the external shell, and the total mass of the robot is 1.86 kg. The ballasts were positioned to vertically align the center of mass with the center of buoyancy and avoid pitch or roll rotations while the robot is still. The buoyancy of the robot is not actively controlled; however, since the robot does not receive any hydrostatic force, it maintains its depth when it is still. Upward and downward motions are obtained with an asymmetric fin movement, as explained in the following section.

### 2.6. Robot Control

**Figure 8:**
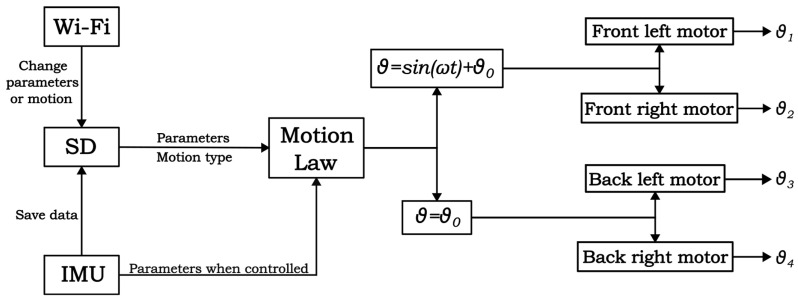
Block diagram of the robot control.

In Figure 8, a block diagram of the control algorithm is presented. The kinematic parameters of the motion law and the type of motion that can be rectilinear or a type of maneuver are communicated by the user through the Wi-Fi when the robot is out of water. These data are stored in an SD-card memory, which the MCU reads when the robot is switched on. Then, a motion law is computed for every motor according to the kinematic parameters previously communicated, and it is executed by the servomotors. For the preliminary tests, without feedback control, the motion law of the pectoral fin motors is a sinusoidal motion law, which can have a mean value different from zero, whereas the caudal fins are just kept still by the motors at the desired angle. The measurement from the gyroscope and the accelerometer are saved in the SD card to allow post-processing of the data, and they are used for real-time estimation of the robot’s orientation.

## 3. Experimental Results

### 3.1. Fin Deformation Assessment

**Figure 9:**
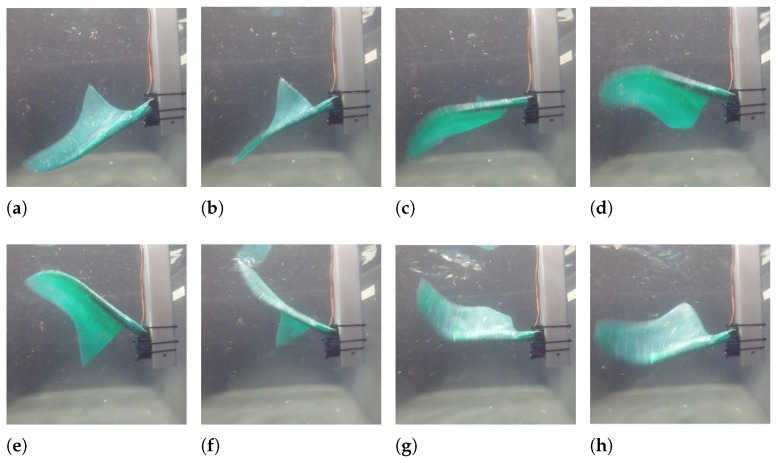
Underwater movement of the fin fixed on a rigid bar. (**a**) 0≡T; (**b**) T/8; (**c**) T/4; (**d**) 3/4 T; (**e**) T/2; (**f**) 5/8 T; (**g**) 3/4 T; (**h**) 7/8 T.

The underwater behavior of the fin is observed in a small tank, and an aluminum structure holds the fin and the motor inside the tank. The motor performs a sinusoidal movement of $\pm 45^{\circ }$, and the corresponding movement of the fin is shown in Figure 9.

The fin movement pushes backward the surrounding water generating a perceivable thrust force on the aluminum structure, and observing the images in Figure 9, it is possible to appreciate that the realized fin moves similarly to its biological reference and that there is a considerable fin tip delay, caused by the very low stiffness of the fin tip.

### 3.2. Swimming Performance Evaluation

The robot was tested underwater in a lake, and a camera was used to capture the fin’s deformation and the robot’s forward movement to assess its swimming performance. During swimming, the principal movement of the robot is the flapping of the pectoral fins, which provide the propulsive force and the moments necessary for maneuvers. Instead, the caudal fins are just used to correct the robot’s orientation, and they are supposed to make small movements.

The objective of these preliminary tests is to evaluate the swimming dynamics of the robot for different movements of the pectoral fins. The swimming velocity is evaluated in post-processing by analyzing the videos recorded with an external camera, whereas the robot’s orientation is computed by combining the measurements of the accelerometer and the gyroscope using a Kalman filter, following the approach of Roetemberg et al. [49]. Due to the small dimensions of the central box of the robot, the magnetometer is close to the electronic board and to the cables that bring the electric current to the motors, which produce very strong soft-iron effects. Moreover, accurate calibration of the magnetometer to counterbalance these disturbances cannot be performed since the electric current is variable with time. Therefore, the magnetometer measurements have not been included in the sensor fusion algorithm, and only gyroscope and accelerometer measurements are used. As a result, the resulting yaw angle is not computed with respect to the Earth’s absolute reference system but with respect to a reference system in which the yaw rotation is null when the robot is turned on.

During the tests, the rotation θ of the leading edge of the pectoral fins is described by Equation (8),

(8) $$\theta \left(t\right)=\theta_{0}+A\sin\left(2\pi ft\right)$$

where $\theta_{0}$ is the mean angle, *A* is the amplitude of the motion, and *f* is the frequency.

**Figure 10:**
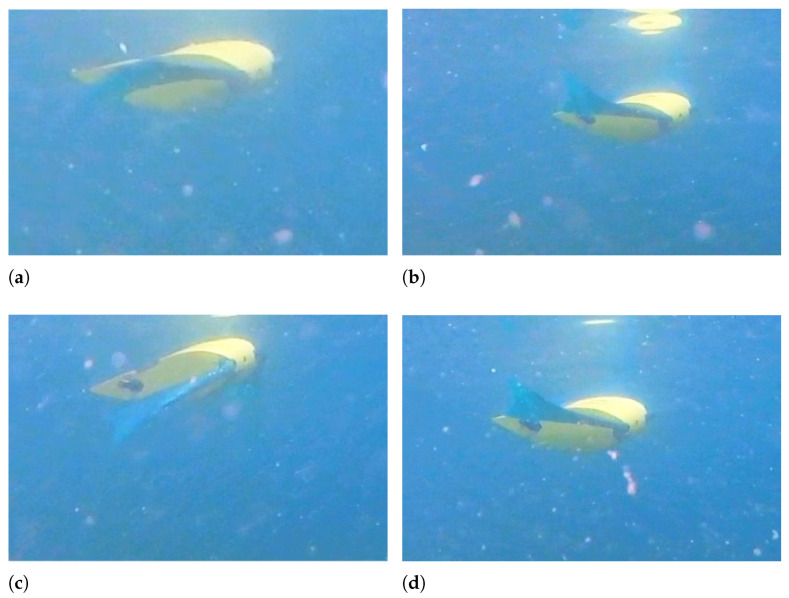
Rectilinear swimming—f = 0.5 Hz - A = 20°. (**a**) t = 2 s; (**b**) t = 2.8 s; (**c**) t = 3.6 s; (**d**) t = 4.4 s.

In Figure 10, a sequence of photograms showing the rectilinear movement of the robot is presented. The pectoral fins are flapping with an equal amplitude of 20°, and the caudal fins are still. When the flapping frequency is 0.5 Hz, the robot moves along a rectilinear trajectory with an average velocity of 0.15 m/s, which corresponds to 0.6 BL/s, and with a frequency of 1 Hz the robot is able to reach a velocity of 0.4 m/s, which corresponds to 1.5 BL/s. These values of velocity are not a result of a direct velocity measurement or of a real-time estimation, but they correspond to the average speed obtained by measuring the elapsed time and the traveled distance for every test.

**Figure 11:**
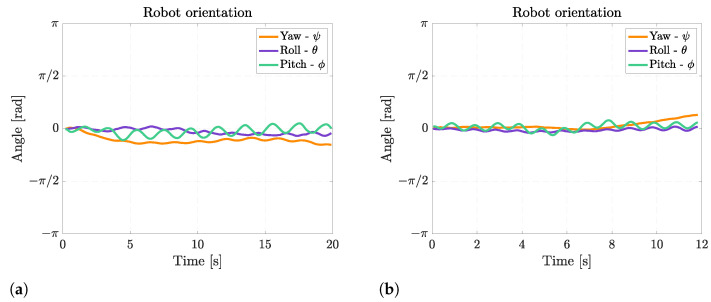
Euler’s angles during rectilinear motion at different fin motion frequencies. (**a**) Rectilinear motion at 0.5 Hz; (**b**) Rectilinear motion at 1 Hz.

During rectilinear forward swimming, the robot slightly rotates about the pitch axis because, when the fins move upward, they also generate a downward-directed force, and when they move upward, they generate an upward-directed force. These forces generate alternate pitching moments on the robot that cause a periodical pitching rotation, as shown in Figure 11. The frequency of pitching rotation is the same as the frequency of fin motion, and the average amplitude of this rotation is 24° at 0.5 Hz and 16° at 1 Hz. The oscillations about the roll axis are of minor importance, and they are caused by small asymmetries between the movements of the left and the right fins.

**Figure 12:**
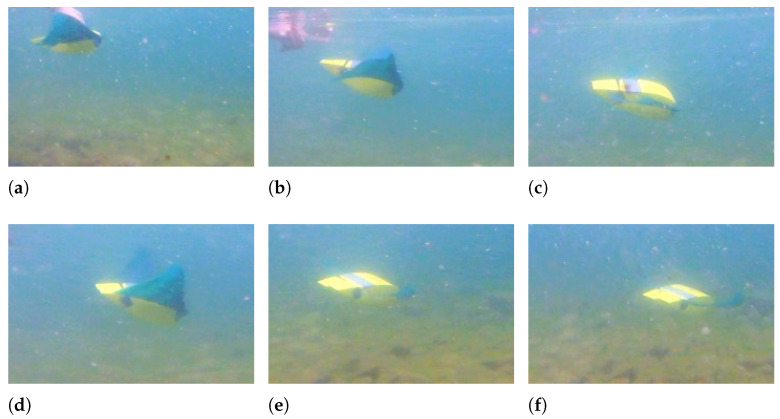
Downward swimming—f = 1 Hz - $\theta_{0}$ = +22.5° - A = 20°. (**a**) t = 0 s; (**b**) t = 0.3 s; (**c**) t = 0.7 s; (**d**) t = 1 s; (**e**) t = 1.5 s; (**f**) t = 2 s.

**Figure 13:**
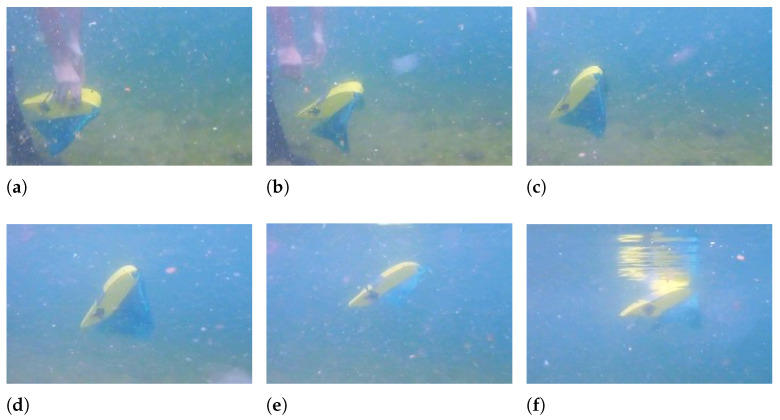
Upward swimming—f = 1 Hz - $\theta_{0}$ = −22.5° - A = 20°. (**a**) t = 0 s; (**b**) t = 0.3 s; (**c**) t = 0.6 s; (**d**) t = 1.2 s; (**e**) t = 2 s; (**f**) t = 2.4 s.

When the fin movement is symmetric with respect to the horizontal plane, the robot follows a horizontal path; conversely, if the fin movement is asymmetric, a pitching moment is produced and floating or diving maneuvers can be achieved. If the mean value of the angle of the fin with respect to the horizontal plane is positive, the robot moves downward, as shown in Figure 12, and when the mean angle is negative, it moves upwards, as shown in Figure 13. In both tests, the frequency of fin flapping is 1 Hz, the amplitude is 20°, and the asymmetry is ±22.5°.

**Figure 14:**
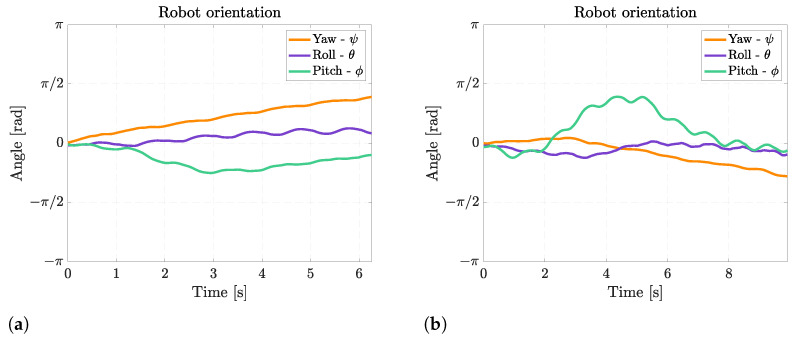
Euler’s angles during diving and floating maneuver. (**a**) Diving maneuver; (**b**) Floating maneuver.

The Euler’s angles of the robot during floating and diving maneuvers are displayed in Figure 14. It can be observed that the robot can achieve a very large pitch rotation of 45° while going downwards and of 65° while going upwards. This difference is caused by the asymmetry in mass distribution between the robot’s front and rear, which makes pitching upward easier than downward. Moreover, the fin movement causes the same small oscillations about the pitch axis as rectilinear swimming.

**Figure 15:**
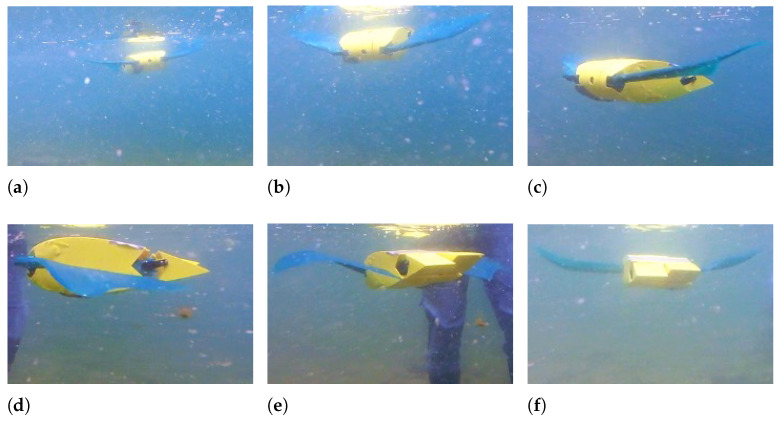
Right turn—f = 1 Hz - $A_{l}$ = 20° - $A_{r}$ = 10°. (**a**) t = 2 s; (**b**) t = 2.5 s; (**c**) t = 3 s; (**d**) t = 3.5 s; (**e**) t = 3.7 s; (**f**) t = 4 s.

**Figure 16:**
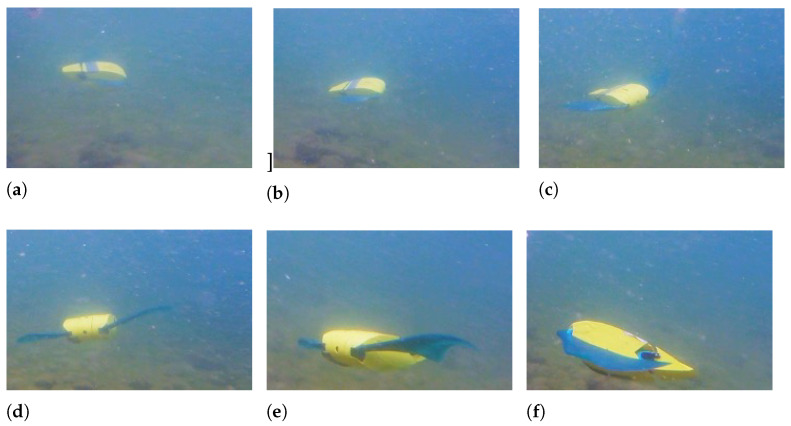
Right turn—f = 1 Hz - $A_{l}$ = 20° - $A_{r}$ = 0°. (**a**) t = 1 s; (**b**) t = 1.5 s; (**c**) t = 2 s; (**d**) t = 2.5 s; (**e**) t = 3 s; (**f**) t = 3.5 s.

To turn left or right, the two fins have a different amplitude of motion, and the larger this difference is, the smaller the curvature radius. In Figure 15 and Figure 16, a right turn is achieved, with the left fin moving with an amplitude of 20° and the right fin with an amplitude of 10° in Figure 15 and 0° in Figure 16.

Moving just one of the two fins, a smaller curvature radius can be achieved, and although the motor connected to the right fin is not active, the right fin still moves and is deformed by the interaction with water producing a traveling wave because of the robot’s periodic pitch and roll rotations caused by the left flapping fin.

**Figure 17:**
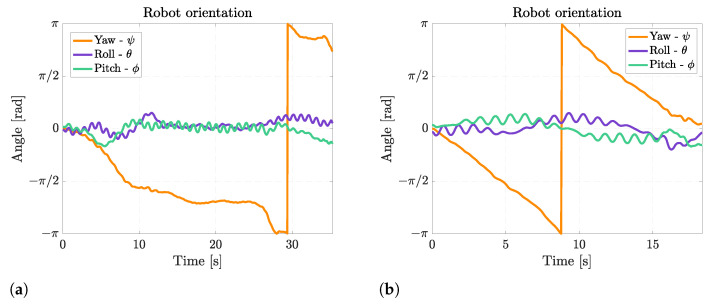
Euler’s angles during right turns. (**a**) Right turn with right fin moving with half amplitude; (**b**) Right turn with right fin still.

Figure 17 shows the Euler’s angles during right-turning maneuvers. The angular velocity about the yaw axis for the right turn with a fin still is 0.36 rad/s, whereas when the right fin moves with half amplitude, the average angular velocity is 0.12 rad/s. When both fins are moving, the angular speed is not constant; this occurs because the robot is also swimming with a non-negligible forward velocity, so the interaction of the fins with the surrounding water is more complex, causing also drifts and lateral movements of the robot. During turning, the same oscillations about the pitch axis occurring in rectilinear swimming can be observed, and the asymmetry between the left and the right fins causes an oscillation about the roll axis too, which has an average amplitude of 15°.

**Figure 18:**
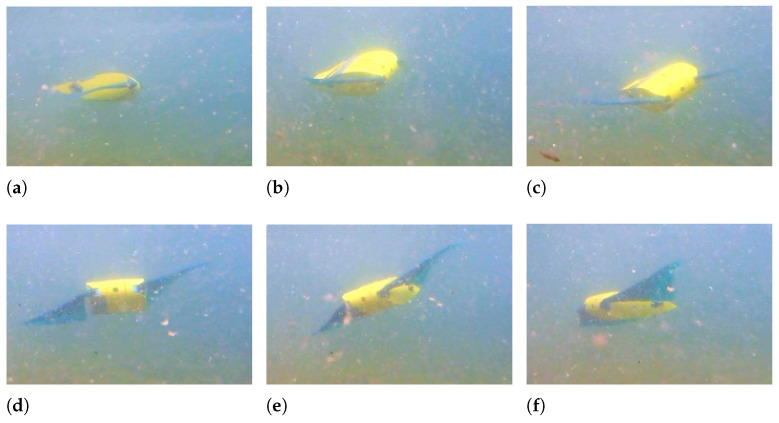
Small curvature radius right turn—f = 1 Hz - $A_{l}$ = 20° - $A_{r}$ = −20°. (**a**) t = 1 s; (**b**) t = 1.5 s; (**c**) t = 2 s; (**d**) t = 2.5 s; (**e**) t = 3 s; (**f**) t = 3.5 s.

A tiny curvature radius can be achieved by moving the two fins with opposite amplitude, as shown in Figure 18, where the left fin has an amplitude *A* of 20°and the right fin of −20°.

**Figure 19:**
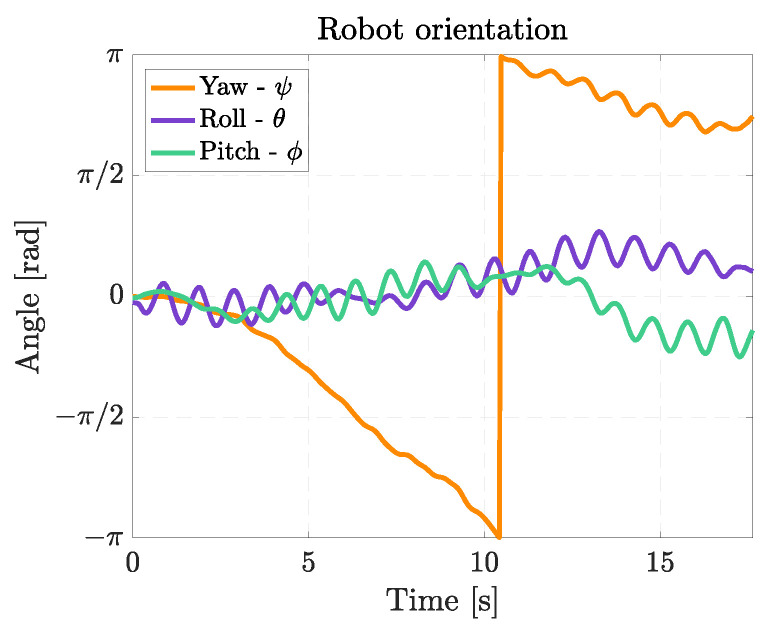
Euler’s angles during a turn with null curvature radius.

When the robot rotates by moving its fins in counter-phase, an average angular speed of 0.32 rad/s can be reached, as shown in Figure 19. The rotation velocity is approximately the same as for the turn with one fin still, but the advantage of this maneuver is the extremely small curvature radius. In this case, the robot oscillates about the pitch axis, and the oscillations about the roll axis are considerable since they reach 33°.

## 4. Discussion

In this work, a biomimetic robot inspired by the cownose ray has been developed, which propels by flapping its pectoral fins. This propulsion mechanism gives these fish great maneuverability and is considered one of the most efficient swimming strategies. These characteristics are due to the fins’ particular shape and movement, which consists of a traveling wave that pushes the surrounding water backward. The fins’ shape accurately reproduces its natural counterpart; however, the pectoral fins of the robot are attached to the main body only at the leading edge, which is actuated by a servomotor. The traveling wave is generated passively by the interaction with water, and leaving the trailing edge detached from the central body increases the flexibility of the fin, allowing it to perform a movement of greater amplitude. The experimental tests have shown that this approach to improving fin flexibility, conceived by Chew et al. [38], is effective since the robots’ fins deform like cownose ray’s fins and generate propulsive thrust.

The swimming tests have demonstrated that this robot, when the fins move at 1 Hz, can reach a velocity of 0.4 m/s, corresponding to 1.5 BL/s; this speed is comparable to the performances of other similar robots moving like batoid fishes, and the normalized speed with respect to the body length is one of the highest among manta and ray robots, as shown in Table 1.

The robot’s maneuverability was assessed, evaluating the robot’s ability to carry out floating and diving maneuvers, which are achieved by asymmetric fin flapping, and turns. These lasts are performed by moving left and right fins with different amplitudes, and the highest this difference, the smaller the curvature radius, and when this difference is maximum, the fins move in counter-phase, and the robot can turn, achieving an almost null curvature radius. Therefore, despite the simplicity of fin actuation and the small number of actuators used, the robot displays excellent maneuverability. Moreover, the robot is equipped with a caudal fin composed of two small rudders that, in future work, will be used to improve maneuverability further and counteract the alternate pitching moment during rectilinear forward swimming.

The robot has shown that the propulsion mechanism is valid and very promising and that it is advantageous to use this kind of fin propulsion for underwater robots that require long endurance, such as those employed for seabed exploration.

In conclusion, in this work, it has been shown that this robot can generate high propulsive thrust and move with great agility in all directions. Future developments of this robot will mainly focus on implementing a control algorithm, allowing it to follow a trajectory and maintain a constant orientation.

## Figures and Tables

**Table 1:** Review of the swimming performances of a variety of biomimetic robots inspired by batoid fishes.

Reference	Body Length [cm]	Speed [m/s]	Speed [BL/s]	Frequency [Hz]	Mass [kg]
Festo (2007) [30] (Aqua Ray)	61.5	0.5	0.81	-	10
Gao et al. (2007) [39]	65	0.4	0.61	0.8	0.6
Cai et al. (2010) [22] (Robo-ray II)	56	0.16	0.28	1.2	3.8
Cai et al. (2010) [22]	40	0.36	0.9	2	3.5
Low et al. (2011) [20] (Ro-Man II)	50	0.4	0.8	1.5	7.3
Low et al. (2011) [20] (Ro-Man III)	37	0.3	0.81	1.5	5
Chen et al. (2011) [40]	21	0.0071	0.034	0.157	0.119
Liu et al. (2015) [19]	43	0.35	0.81	1.82	-
Ma et al. (2015) [28]	40.4	0.43	0.94	1	4.6
Chew et al. (2015) [38]	28	0.5	1.78	0.9	0.77
Li et al. (2017) [17]	9.3	0.064	0.69	5	0.09
Zhang et al. (2018) [29]	48.5	0.4	0.82	2.5	6
He et al. (2020) [25]	40.1	0.32	0.8	1	4.3
Meng et al. (2020) [26]	38.1	0.37	1	1	3.68
Hao et al. (2022) [24]	80	0.8	1	0.5	7
Chen et al. (2022) [32]	58	0.68	1.17	0.8	8
Proposed robot	26	0.4	1.5	1	1.86

## Data Availability

Not applicable.

## Abbreviations

The following abbreviations are used in this manuscript:

AUV	Autonomous Underwater Vehicle
BCF	Body-Caudal Fin
MPF	Median Paired Fin
IMU	Inertial Measurement Unit
LiPo	Lithium Polymer
CAD	Computer-Aided Design
FEA	Finite Element Analysis
BL	Body Length
